# Time to first antenatal care visit and its predictors among women in Kenya: Weibull gamma shared frailty model (based on the recent 2022 KDHS data)

**DOI:** 10.1186/s12884-025-07178-y

**Published:** 2025-01-22

**Authors:** Bizunesh Fantahun Kase, Beminate Lemma Seifu, Kusse Urmale Mare, Abdu Hailu Shibeshi, Hiwot Altaye Asebe, Kebede Gemeda, Zufan Alamrie Asmare, Yordanos Sisay Asgedom, Bezawit Melak Fente, Afework Alemu Lombebo, Tsion Mulat Tebeje

**Affiliations:** 1 Department of Public Health, College of Medicine and Health Sciences, Samara University, Samara, Ethiopia; 2 Department of Nursing, College of Medicine and Health Sciences, Samara University, Samara, Ethiopia; 3 Department of Statistics, College of Natural and Computational Science, Samara University, Samara, Ethiopia; 4 Department of Ophthalmology, School of Medicine and Health Science, Debre Tabor University, Debre Tabor, Ethiopia; 5 Department of Epidemiology and Biostatistics, College of Health Sciences and Medicine, Wolaita Sodo University, Sodo, Ethiopia; 6 Department of General Midwifery, School of Midwifery, College of Medicine & Health Sciences, University of Gondar, Gondar, Ethiopia; 7 School of Medicine, College of Health Science and Medicine, Wolaita Sodo University, Sodo, Ethiopia; 8 School of Public Health, College of health sciences and Medicine, Dilla University, Dilla, Ethiopia

**Keywords:** First antenatal care visit, Kenya, Weibull gamma shared frailty model

## Abstract

**Background:**

The first trimester of pregnancy is critical for fetal development, making early antenatal care visits essential for timely check-ups and managing potential complications. However, delayed antenatal care initiation remains a public health challenge in sub-Saharan Africa, including Kenya. Therefore, this study aimed to assess and provide up-to-date information on time to first antenatal care visit and its predictors among women in Kenya, using data from the most recent 2022 Kenya Demographic and Health Survey (KDHS).

**Methods:**

This community-based cross-sectional study analyzed data from 19,530 birth histories in the 2022 Kenya Demographic and Health Survey (KDHS). The primary outcome was the timing of the first antenatal care (ANC) visit, classified as timely if it occurred in the first trimester. Shared frailty survival models were used to account for the hierarchical data structure and unobserved heterogeneity, with the Weibull gamma model identified as the best fit based on Information Criteria (AIC), and Bayesian Information Criteria (BIC). Variables with *p* < 0.2 entered multivariable analysis, and results were reported as Adjusted Hazard Ratios (AHR) with 95% Confidence Intervals (CI) using the Weibull gamma model.

**Results:**

The study found that the median time for the first antenatal care (ANC) visit in Kenya was four months. Significant predictors of ANC timing included women’s age (35–49 years: AHR 0.83; 95% CI: 0.72–0.95), education level (higher: AHR 1.45; 95% CI: 1.17–1.78), media exposure (yes: AHR 1.21; 95% CI: 1.05–1.39), parity (four or more children: AHR 0.81; 95% CI: 0.72–0.91), wealth status (richest: AHR 2.00; 95% CI: 1.63–2.43), desire for more children (did not want more: AHR 0.64; 95% CI: 0.54–0.77), residence (rural: AHR 1.22; 95% CI: 1.07–1.39), and religion (Islam: AHR 0.76; 95% CI: 0.64–0.89).

**Conclusion:**

The median time for the first ANC visit exceeds the World Health Organization’s recommendation of initiating care within the first trimester. These findings underscore the need for targeted interventions to promote timely ANC, especially among women with limited media exposure, high parity, lower socioeconomic status, and specific religious followers.

**Supplementary Information:**

The online version contains supplementary material available at 10.1186/s12884-025-07178-y.

## Background

Antenatal care (ANC) is the care provided by skilled healthcare professionals to pregnant women and adolescent girls to ensure the best possible health outcomes for both the mother and the child during pregnancy. The components of antenatal care (ANC) are comprehensive, including risk assessment, prevention, detection, and treatment of pregnancy-related problems, as well as health promotion and education [1]. According to the 2016 WHO ANC framework, the first antenatal care visit should occur within the first trimester, or 12 weeks of gestation. This early visit is crucial as it provides an opportunity for the treatment and prevention of current and potential causes of morbidity and mortality in both mothers and newborns. In addition to providing timely and relevant information and support during pregnancy, this approach aims to offer pregnant women an integrated package of high-quality care based on optimal evidence-based practices [2, 3].

Delayed initiation of antenatal care (ANC) visits remains a significant public health challenge, with profound implications for the quality of care a pregnant woman receives. The late timing of the initial ANC visit can negatively impact the timely detection and management of pregnancy-related complications, access to essential health services, provision of health education, and overall maternal and neonatal health outcomes [4]. This delay contributes to the “obstetric transition,” a shift in the pattern of obstetric outcomes due to increased mortality and morbidities linked to pre-existing medical conditions [5].

Maternal mortality and stillbirths remain the greatest global health concern, despite improvements in the use of maternal health services globally. According to the World Health Organization (WHO), the MMR declined by approximately 34% between 2000 and 2020, from 339 deaths per 100,000 live births to 223 deaths per 100,000 live births [6]. Despite this progress, maternal mortality remains a critical issue, with around 287,000 women dying from complications related to pregnancy and childbirth in 2020 [7]. Sub-Saharan Africa bears the majority of this burden, accounting for about 70% of global maternal deaths in 2020, with an MMR of 545 per 100,000 live births [6].

In Kenya, the maternal mortality rate (MMR) also remains significantly high [8]. Despite efforts by the government to enhance maternal health through initiatives such as supplying medical resources, training healthcare workers, and establishing facilities offering perinatal care, significant challenges persist. Additionally, operational and management-level policy guidelines have been introduced to tackle these issues [9]. Recent estimates indicate that Kenya’s MMR was approximately 530 deaths per 100,000 live births in 2020, marking a slight increase from previous years and underscoring persistent challenges in maternal healthcare [10]. The primary causes of maternal deaths during pregnancy are preventable obstetric complications, including obstetric hemorrhage, hypertensive disorders (such as pre-eclampsia and eclampsia), unsafe abortions, and infections [11].

The Sustainable Development Goals and broader strategies for improving maternal health programs were outlined in the 2015 Strategies for Ending Preventable Maternal Mortality (EPMM). These strategies aim to address critical gaps that lead to disparities in access, quality, and treatment outcomes both within and across countries. Grounded in a human rights approach to maternal and newborn health, the EPMM highlights the critical role of improving and ensuring timely antenatal care in significantly reducing maternal mortality and advancing progress toward these goals [12]. Addressing these challenges through enhanced antenatal care is essential for achieving the goals outlined in the EPMM strategies [13].

Previous studies have identified several predictors of the timing of the first antenatal care (ANC) visit. These include the educational status of women [14–16], level of household wealth index [14, 15], community women literacy, distance to health facility [14], having media exposure [14, 15, 17], residence [16], husband education, husband occupation [15]. Data from the Kenya Demographic and Health Survey (KDHS) indicate that while overall ANC coverage is relatively high, many women attend their first visit later in pregnancy [18]. To date, only a limited number of similar studies have been conducted in Kenya. Most existing research focuses on the number of ANC visits during pregnancy and the proportion of women delaying their first visit, often limited to specific districts and lacking recent data [4, 19–21]. Understanding the timing of the first ANC visit is crucial for providing program planners and policymakers with the necessary insights to mitigate the adverse effects of delayed ANC visits.

This study utilized data from the 2022 Kenya Demographic and Health Survey (KDHS) to provide up-to-date information on the time to first antenatal care (ANC) visit. To address limitations in previous studies, a parametric shared frailty model was applied to analyze the time to the first ANC visit and its determinants. This model was employed to allow a comprehensive analysis of predictors of the time to the first ANC visit, accounting for both individual-level factors and shared frailty within communities. This approach provides a deeper understanding of the underlying determinants contributing to delayed ANC attendance among women in Kenya. Therefore, this study aimed to assess time to first antenatal care visit and its predictors among women in Kenya. By quantifying the magnitude of the problem and identifying its predictors, this study seeks to inform targeted interventions and policies aimed at promoting timely access to ANC services and improving maternal and child health outcomes in Kenya.

## Methods

### Data source, study design and setting

This study was conducted in Kenya using data from the recent Kenya Demographic and Health Survey (KDHS 2022), a publicly accessible and nationally representative survey. The data was collected through a community-based cross-sectional design.

### Study population, and sampling technique

Sampling for the study was in two stages. In the first stage, 1,692 clusters were independently selected from each sampling stratum in the K-HMSF using equal probability. In the second stage, 25 households were randomly selected from each cluster, resulting in a total sample of 42,300 households. Among these, 19,530 women aged 15–49 years were interviewed.

**Fig. 1:**
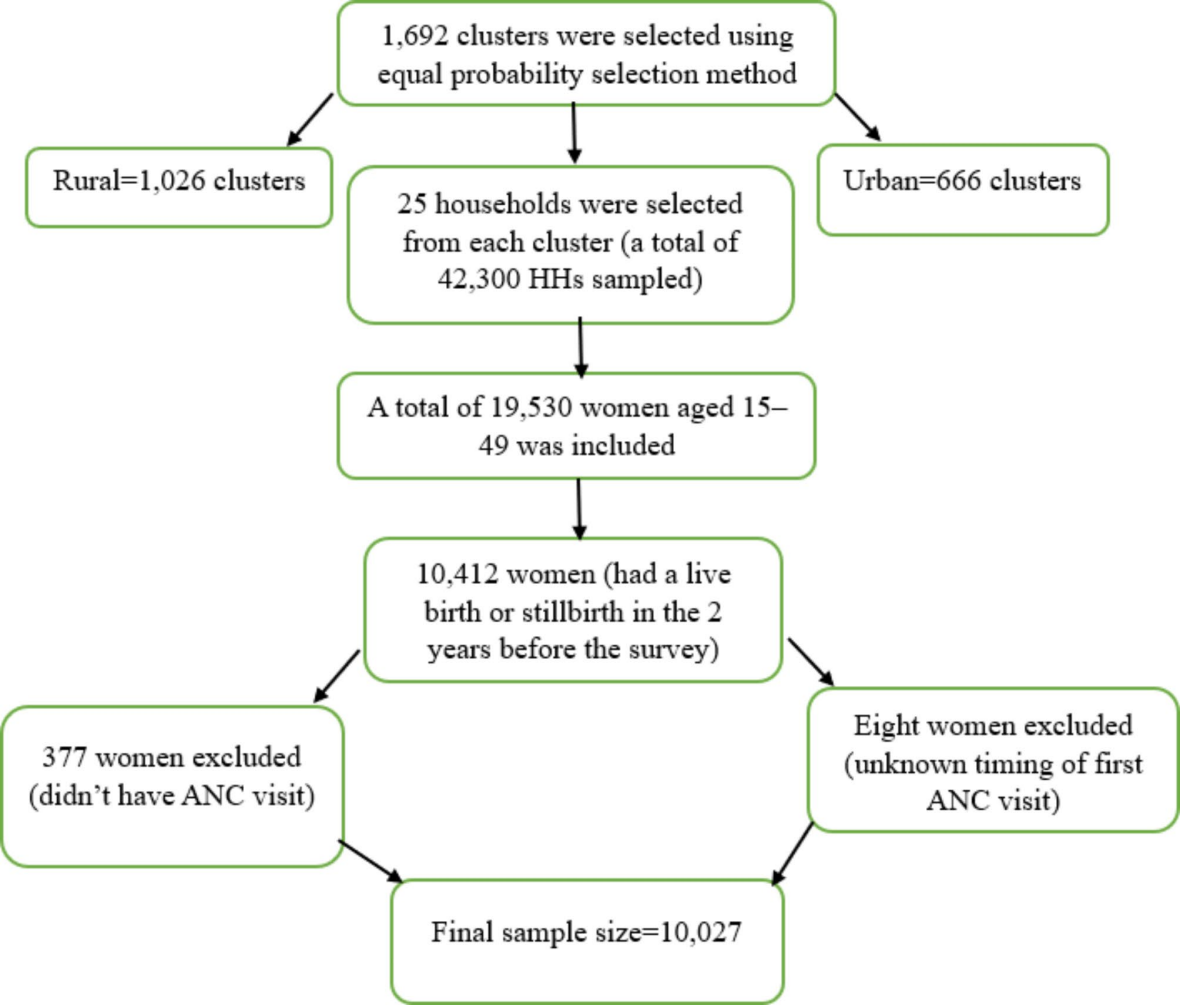
Flowchart of the sampling procedures for the study on time to first ANC visit and its predictors among women in Kenya, KDHS, 2022

From this group, 10,412 women aged 15–49 who had a live birth or stillbirth in the two years preceding the survey were interviewed about antenatal care (ANC) services received from skilled providers. Of these, 377 women who did not have any ANC visits and 8 women who did not know the gestational age during their first ANC visit were excluded, leaving a final sample size of 10,027 women for this study (Fig. 1).

### Inclusion and exclusion criteria

The study included women aged 15 to 49 who had a live birth or stillbirth within the two years preceding the survey and attended at least one antenatal care visit during that period. Women with unknown gestational age at their first ANC visit were excluded.

### Study variables and measurements

#### Dependent variable

The outcome variable of this study was the time to first antenatal care (ANC) visit, measured in months from conception to the first ANC visit for women who attended at least one ANC session.

#### Event

A woman’s first ANC visit was classified as timely (event) if it occurred during the first trimester; otherwise, it was considered delayed (censored).

### Independent variables

Explanatory variables were selected based on a literature review, identifying factors associated with the timing of the first ANC visit. These variables included the woman’s age, educational level, parity, media exposure, sex of the household head, wealth status, marital status, distance to the health facility, desire for the last child, husband or partner’s education, religion, and place of residence. Two additional community-level variables were derived by aggregating individual-level data at the cluster level, as they were not directly available in the Demographic Health Survey dataset. The aggregates were calculated using the average proportions of women in each category of the respective variables. Median values were then used to classify these aggregated variables into categories, such as low and high community-level women’s illiteracy and media exposure (Table 1).

**Table 1:** List of independent variables for the study on time to first ANC visit and its predictors among women in Kenya, KDHS, 2022

Variables	Description/Category
Age	The age of the mother was categorized as 15–24,25–34, 35–49
Media exposure	women exposed to one of the media (reading newspapers/magazine, listening to the radio, watching television) was classified as yes/no
Parity	The number of children ever born and is categorized as 3 or less and 4 or more
Educational status	Women’s educational status was categorized as No education, Primary, Secondary and higher
Sex of the household head	Categorized as male/female
Wealth status	It was categorized as Poorest, Poorer, Middle, Richer, and Richest
Marital status	Categorized as currently in union/not in union
Distance to the health facility	Distance to a health facility as a major problem in accessing health care for themselves was classified big problem/not a big problem
Wanted last-child	Women wanted their last birth; categorized as wanted then, wanted later, and wanted no more.
Husband/partner education (*n* = 13,695)	Husband/partner education; categorized as No education, Primary, Secondary, and higher
Religion	Respondent’s religion is classified as Protestant, Evangelical church, Catholic, Islam, and Others
Residence	The Respondent’s place of residence was Urban/Rural
Community-level illiteracy	Measured as the proportion of women in a cluster with a high/low percentage of women without formal education.
Community-level media exposure	Measured as the proportion of women in a cluster with a high/low percentage of women with no exposure to either newspaper/magazine, radio or television, or both.

### Data management and analysis

Data analysis was conducted using STATA version 17. Descriptive statistics, including unweighted frequencies and weighted percentages, were used to present the distribution of women according to their characteristics. The dataset was weighted using the v005 variable, which represents the sampling weight. For analysis, this variable was divided by 1,000,000 to ensure the data accurately reflected the population structure. Variations in survival distributions across different variable categories were compared using the log-rank test. Survival time was analyzed using the Kaplan-Meier estimation method. The proportional hazard (PH) assumption was tested using the Schoenfeld residuals test. The assumption was found to be violated, as indicated by a global p-value of less than 0.05 (Table 2).

Considering the hierarchical nature of the DHS data and to account for unobserved heterogeneity, we fitted shared frailty survival models. By using the frailty model (random effect survival model), we assessed the existence of clustering. The presence or absence of significant clustering was evaluated using the theta parameter, which was significant at the null model (θ = 0.26, 95% CI: 0.21, 0.33). According to the likelihood ratio (LR) test for theta = 0 (X² = 129.81, *p* < 0.001), there was unobserved heterogeneity or shared frailty, indicating that women in a cluster had a higher risk of being connected with other women in the same cluster.

The best-fitting model was selected based on the Akaike Information Criterion (AIC) and Bayesian Information Criterion (BIC), with the model having the lowest values of these criteria considered the best fit. In the shared frailty model, enumeration areas (EAs)/clusters were used as random effects. The baseline and frailty distributions included gamma and inverse Gaussian, with baseline hazard functions modeled using Weibull, Gompertz, Exponential, log-logistic, and lognormal distributions. Among these, the Weibull gamma shared frailty model demonstrated the best fit, as indicated by the lowest AIC and BIC values (Supplemental file 1).

In the bivariable Weibull gamma shared frailty analysis, variables with a p-value of less than 0.20 were included in the multivariable analysis. The analysis estimated the 95% confidence interval and hazard ratio. Significant predictors of time to first ANC visit were identified in the multivariable analysis using the Adjusted Hazard Ratio (AHR) with a 95% Confidence Interval (CI).

**Table 2:** Schoenfeld residual test for assessing the proportional hazards assumption for the study on time to first ANC visit and its predictors among women in Kenya, KDHS, 2022

Variables	rho	chi2	df	Prob > chi2
Age of the women	0.03697	1.65	1	0.1993
Media exposure	0.03500	1.63	1	0.2014
Parity	0.01123	0.15	1	0.6989
Educational level	0.01550	0.27	1	0.6010
Sex of household head	0.00403	0.02	1	0.8898
Wealth index	0.03349	1.23	1	0.2680
Distance to health facility	0.03855	1.70	1	0.1920
Wanted last child	0.04935	2.97	1	0.0847
Husband educational level	0.00346	0.01	1	0.9095
Religion	0.02256	0.65	1	0. 4207
v025	0.00547	0.03	1	0.8526
Community level illiteracy	0.03783	1.76	1	0.1851
Community level media exposure	0.03314	1.18	1	0.276 4
**Global test**		**22.86**	**13**	**0.0434**

## Results

### Descriptive statistics

The study included 10,027 women who had experienced a live birth or stillbirth within the two years prior to the survey and had a history of ANC follow-up during pregnancy. Among them, 48.51% were aged 25–34 years, and 62.27% resided in rural areas. Regarding marital status, 79.07% of the women were in a union. Additionally, 36.77% had completed only secondary education (Table 3).

**Table 3:** Socio-demographic and obstetric characteristics, and log-rank test for the study on time to first ANC visit and its predictors among women in Kenya, KDHS, 2022

Variable	Categories	Unweighted frequency (Weighted Percentage in %)	First ANC visit status		Log-rank test p-value
			Unweighted censored (weighted %)	Unweighted event (weighted %)	
Age	15–24	3,363 (33.96)	2,416 (69.85)	947 (30.15)	*<* 0.001
	25–34	4,735 (48.51)	3,357 (67.24)	1,378 (32.76)	
	35–49	1,929 (17.52)	1,472 (72.73)	457 (27.27)	
Media exposure	Yes	7,767 (86.25)	5,417 (67.52)	2,350 (32.48)	*<* 0.001
	No	2,260 (13.75)	1,828 (78.95)	432 (21.05)	
Marital status	Currently in union	8,118 (79.05)	5,845 (68.59)	2,273 (31.41)	0.2175
	Currently not in union	1,909 (20.95)	1,400 (70.98)	509 (29.02)	
Parity	3 and less	6,738 (72.44)	4,647 (66.15)	2,091 (33.85)	*<* 0.001
	4 and above	3,289 (27.56)	2,598 (76.82)	691 (23.18)	
Educational status	No education	1,834 (8.26 )	1,463	371	*<* 0.001
	Primary	3,436 (35.55)	2,587	849	
	Secondary	3,238 (36.77)	2,352	886	
	Higher	1,519 (19.42)	843	676	
Sex of the household head	Male	7,064 (71.19)	5,084 (68.38)	1,980 (31.62)	0.3371
	Female	2,963 (28.8)	2,161 (70.85)	802 (29.15)	
Wealth status	Poorest	2,988 (20.97)	2,346 (78.07)	642 (21.93)	*<* 0.001
	Poorer	1,717 (17.69)	1,278 (73.23)	439 (26.77)	
	Middle	1,798 (17.80)	1,339 (72.78)	459 (27.22)	
	Richer	2,010 (21.29)	1,436 (70.16)	574 (29.84)	
	Richest	1,514 (22.26)	846 (53.36)	668 (46.64)	
Distance to the health facility(*n* = 5,256)	Big problem	1,615 (25.54)	1,204 (73.01)	411 (26.99)	0.0758
	Not a big problem	3,641 (74.46)	2,626 (70.01)	1,015 (29.99)	
Wanted last child	Wanted then	6,469 (60.48)	4,579 (66.54)	1,890 (33.46)	*<* 0.001
	Wanted later	2,778 (30.66)	2,039 (71.35)	739 (28.65)	
	Wanted no more	780 (8.86)	627 (78.69)	153 (21.31)	
Husband/partner education (*n* = 8,043)	No education	1,479 (8.33)	1,187 (79.72)	292 (20.28)	*<* 0.001
	Primary	2,680 (34.74)	2,004 (73.49)	676 (26.51)	
	Secondary	2,282 (32.50)	1,622 (69.38)	660 (30.62)	
	Higher	1,602 (24.43)	969 (56.16)	633 (43.84)	
Religion	Protestant	3,215 (36.36)	2,242 (67.00)	973 (33.00)	*<* 0.001
	Evangelical churches	2,111 (24.49)	1,520 (70.76)	591 (29.24)	
	Islam	1,757 (8.02)	1,427 (75.73)	330 (24.27)	
	Catholic	1,765 (18.45)	1,247 (31.29)	518 (68.71)	
	Others	1,179 (12.68)	809 (68.22)	370 (31.78)	
Residence	Urban	3,533 (37.73)	2,425 (63.59)	1,108(36.41)	*<* 0.001
	Rural	6,494 (62.27)	4,820(72.42)	1,674 (27.58)	
Community level illiteracy	High illiteracy	3,014 (15.87)	2,356 (75.49)	658 (24.51)	*<* 0.001
	Low illiteracy	7,013 (84.13)	4,889 (67.88)	2,124 (32.12)	
Community level media exposure	High	3,956	3,088 (76.60)	868 (23.40)	*<* 0.001
	Low	6,071	4,157 (66.27)	1,914 (33.73)	

### Non-parametric survival analysis

**Fig. 2:**
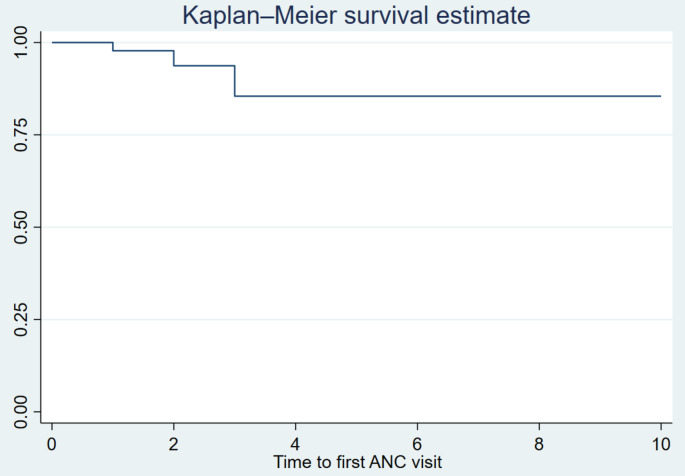
Kaplan-Meier failure curve illustrating time to first ANC visit among women in Kenya, KDHS, 2022

The study found that the median time to the first ANC visit was four months. A non-parametric survival analysis method, the Kaplan-Meier curve, was used to visualize the survival time. The curve revealed an initial sharp decline, followed by a constant rate over the remaining observed period (Fig. 2). This suggests that the likelihood of delaying an ANC visit is higher during early pregnancy and stabilizes as gestational age progresses.

The study also performed a Log-rank test to compare the survival functions across different groups of independent variables. The results indicated a statistically significant difference in survival experiences among the covariates of age, media exposure, parity, educational status, desire for the last child, husband/partner’s education, religion, residence, community illiteracy, and community media exposure (P value < 0.001). This suggests that these covariates significantly influence the time to first ANC visit.

### Predictors of time to first ANC visit in Kenya

The final Weibull gamma shared frailty model identified significant predictors of time to first antenatal care (ANC) visit in Kenya, including women’s age, media exposure, parity, educational level, wealth status, desire for the last child, place of residence, and religion. Compared to women aged 15–24, women aged 35–49 were 17% less likely to initiate their first ANC visit earlier AHR: 0.83; 95% CI: 0.72–0.95). Women exposed to media were 1.21 times more likely to initiate their first ANC visit earlier than those who were not exposed (AHR: 1.21; 95% CI: 1.05–1.39).

Additionally, women with four or more children were 19% less likely to have an early first ANC visit compared to those with three or fewer children (AHR: 0.81; 95% CI: 0.72–0.91). Women with higher levels of education were 1.45 times more likely to initiate their first ANC visit earlier compared to those with no formal education (AHR: 1.45; 95% CI: 1.17–1.78). Women in the highest wealth quintile were twice as likely to have an earlier first ANC visit compared to those in the lowest wealth quintile (AHR: 2.00; 95% CI: 1.63–2.43). Regarding religion, followers of Islam were 24% less likely to have an early first ANC visit compared to Protestant followers (AHR: 0.76; 95% CI: 0.64–0.89). Women who wanted their last child at the time of conception were 36% less likely to have an early first ANC visit compared to those who wanted no more (AHR: 0.64; 95% CI: 0.54–0.77). Finally, rural women were 1.22 times more likely to have an early first ANC visit compared to their urban counterparts (AHR: 1.22; 95% CI: 1.07–1.39) (Table 4).

**Table 4:** The bi-variable and multivariable Gompertz gamma shared frailty model for predictors of time to first ANC visit in Kenya

Variables	Categories	Unadjusted HR (CI: 95%)	Adjusted HR (CI: 95%)
Age	15–24	1	1
	25–34	1.06(0.97, 1.15)	0.99 (0.88, 1.07)
	35–49	0.78 (0.70, 0.88)	0.83(0.72, 0.95) *
Media exposure	No	1	
	Yes	1.65(1.47, 1.85)	1.21(1.05, 1.39) *
Parity	3 and less	1	1
	4 and above	0.61 (0.56, 0.67)	0.81(0.72, 0.91) **
Educational status	No education	1	1
	Primary	1.25(1.09, 1.44)	0.93 (0.78, 1.10)
	Secondary	1.43(1.24, 1.65)	0.87 (0.71, 1.05)
	Higher	2.88(2.48, 3.34)	1.45 (1.17, 1.78) *
Sex of household head	Male	1	
	Female	0.89(0.82, 0.98)	0.93 (0.85, 1.01)
Wealth status	Poorest	1	1
	Poorer	1.17(1.02, 1.33)	1.04 (0.91, 1.21)
	Middle	1.24(1.08, 1.41)	1.05 (0.90, 1.23)
	Richer	1.46(1.28, 1.66)	1.18(0.99, 1.40)
	Richest	2.71(2.38, 3.09)	2.00(1.63, 2.43) **
Wanted last child (*n* = 13,132)	Wanted then	1	1
	Wanted later	0.79(0 0.72, 0.86)	0.79(0.72, 0.87)
	Wanted no more	0.53(0.44, 0.62)	0.64 (0.54, 0.77) **
Religion	Protestant	1	1
	Evangelical churches	0.89 (0.80, 0.99)	0.97(0.87, 1.08)
	Islam	0.58(0.50, 0.67)	0.76(0.64, 0.89) **
	Catholic	0.95(0.85, 1.07)	0.99 (0.83, 1.18)
	Other	1.03 (0.91, 1.18)	1.06 (0.87, 1.29)
Residence	Urban	1	1
	Rural	0.75(0.68, 0.83)	1.22 (1.07, 1.39) **
Community level illiteracy	High literacy	1	1
	Low literacy	1.50 (1.34, 1.69)	0 0.99 (0.84, 1.17)
Community level media exposure	High	1	1
	Low	1.58(1.42, 1.75)	1.14(1.00, 1.30)

## Discussion

The current study found that the median time for the first ANC visit among women in Kenya was four months. This highlights a significant discrepancy from the updated 2016 WHO guidelines, which recommend the first ANC visit within the first 12 weeks (three months) of pregnancy [22]. This finding is consistent with a study conducted in Ethiopia [23], and another in Kenya based on the 1993 KDHS [24]. This delay in the initiation of ANC visits can have critical implications for maternal and neonatal health. Early ANC visits are essential for providing necessary support, particularly in the first trimester, to prevent adverse outcomes for both mothers and newborns. Missing these early opportunities may compromise the health and well-being of both the mother and the fetus, leading to higher risks of complications and adverse outcomes [1].

Another significant implication of this finding is that the median time for the first ANC visit has remained consistent—four months—despite nearly three decades between the 1993 KDHS and the 2022 KDHS. This indicates persistent challenges in maternal healthcare access and utilization in Kenya. This consistency suggests that, despite potential advancements in healthcare infrastructure and public health interventions [25], substantial barriers to early antenatal care have not been effectively addressed over the years.

However, the current findings reveal that women in Kenya had a median time for their first ANC visit earlier than those reported in studies conducted in Ethiopia [26], Nigeria [27], and Tanzania [28]. This variation may be attributed to several factors, such as differences in study periods, healthcare infrastructure, public health policies, and cultural practices across these countries.

Notably, the studies in Ethiopia, Nigeria, and Tanzania utilized data from earlier periods—2016, 2013, and 2012, respectively—while the current study is based on the 2022 KDHS data. The timing of these studies aligns with evolving global guidelines for antenatal care. The WHO updated its recommendations in 2016, advocating for the first ANC visit to occur within the first 12 weeks of pregnancy. While this guideline was introduced around the time of the 2016 EDHS in Ethiopia, it likely required time for integration into public health practices and widespread awareness among healthcare providers and the public.

In contrast, by the time the 2022 KDHS was conducted in Kenya, several years had passed since the guideline update. This additional time likely facilitated the dissemination and implementation of the recommendations in Kenya, contributing to earlier initiation of ANC visits among women.

The study also identified several significant predictors influencing the time to the first ANC visit. These include women’s age, media exposure, parity, educational level, wealth status, desire for the most recent child, religion, and place of residence.

In comparison to women who were not exposed to the media, the first ANC visit occurred earlier for those who were. This finding is supported by studies done in Pakistan [29], Nigeria [30], Ethiopia [26], and Nepal [26]. This finding underscores the crucial role of media as a channel for disseminating health information and raising awareness about the importance of early antenatal care. Media exposure likely enhances knowledge of maternal health services, empowers women to prioritize their health, and influences positive health-seeking behaviors [31]. Additionally, media campaigns may reduce cultural or informational barriers that delay ANC visits [32]. These results highlight the potential of targeted media interventions in promoting early utilization of antenatal services, particularly in regions where access to other sources of health information may be limited [33].

In line with a study in Ethiopia [34], Nigeria [35], and Papua New Guinea [36], compared to women who had three or less children, those who had four or more children had later first ANC visits. This delay may be attributed to several factors, including increased confidence and perceived experience in managing pregnancy among multiparous women, leading to a lower perceived need for early ANC visits [37]. Additionally, competing responsibilities such as childcare and household duties may limit their ability to prioritize early antenatal care [38]. Cultural norms and resource constraints may also play a role, as women with larger families may face financial or logistical challenges in accessing health services. These findings emphasize the need for targeted interventions to encourage timely ANC visits among multiparous women, addressing barriers and reinforcing the importance of early care regardless of previous childbirth experience.

Women who had wanted their last pregnancy were more likely to have their first ANC visit sooner than those who had an unwanted pregnancy. This finding is supported by previous studies [39, 40], This may be the result of the mother’s desire to maintain the baby’s health during her desired pregnancy. As a result, they are eager to receive the follow-up sooner. This highlights the role of maternal attitudes and intentions toward pregnancy in shaping health-seeking behavior. Women with a wanted pregnancy are more likely to prioritize maternal and fetal health, actively seeking early antenatal care to ensure positive outcomes. Conversely, women who did not desire the pregnancy may experience emotional distress, reduced motivation, or a lack of preparedness, potentially delaying engagement with healthcare services [41]. These findings emphasize the importance of strengthening family planning services and providing supportive counseling to address the unique challenges faced by women with unintended pregnancies, fostering earlier and more equitable access to ANC services.

The timing of the first ANC visit was shorter among women with richest wealth status, compared to those with the poorest wealth status, consistent with findings from studies conducted in conducted in Nigeria [35] and Ethiopia [26]. This underscores the significant influence of socioeconomic factors on access to maternal healthcare services. Women with higher wealth status are less likely to face financial barriers, allowing them to afford transportation, healthcare fees, and other related expenses, which facilitates earlier ANC visit [42]. Additionally, they may have better access to healthcare facilities and information, as well as greater autonomy in making health-related decisions.

In contrast, women with lower wealth status may encounter financial constraints, limited access to healthcare resources, and competing priorities that delay ANC visits. These disparities highlight the need for policies and programs aimed at reducing financial and structural barriers, ensuring equitable access to timely antenatal care for women across all socioeconomic strata.

Interestingly, the study found that women residing in rural areas were more likely to have an earlier first ANC visit compared to their urban counterparts, a result that contradicts the findings of most previous studies [43–45]. This unexpected outcome could be influenced by targeted healthcare initiatives or outreach programs in rural areas of Kenya that emphasize early ANC utilization, particularly in settings where maternal health services are prioritized to address historically lower access [46]. Additionally, rural women may perceive antenatal care as more essential due to limited healthcare options and greater reliance on preventive services. In urban settings, despite better accessibility, competing demands, misconceptions about the necessity of early visits, or complacency due to the perceived proximity of services might contribute to delays. This finding highlights the complexity of geographic and contextual factors influencing health-seeking behavior and underscores the need for tailored interventions that address specific barriers in both rural and urban settings.

As compared to followers of protestant religion, Muslims had longer first ANC visit timing. Supported by a study in Nigeria [35] and Ethiopia [47]. Charismatic Christian teams provide free medical care and counseling to its members, may potentially increase the number of women who seek medical attention earlier.

The strengths of our study include the use of a nationally representative survey dataset, enhancing the generalizability of the findings to all women of reproductive age. Additionally, the recent KDHS 2022 dataset provided context-specific insights, such as rural women initiating antenatal care earlier than urban women, reflecting unique healthcare dynamics in Kenya. The study also accounted for clustering effects, addressing correlations within clusters, and incorporated unobserved heterogeneity in the model. However, the study has limitations. Recall bias may have affected the accuracy of mothers’ memories of events that occurred within the survey’s two-year reference period. Furthermore, reliance on secondary data restricted the inclusion of certain important variables that are significant predictors of the time to first antenatal care among Kenyan women.

## Conclusion

This study revealed that the median time for the first antenatal care (ANC) visit in Kenya is four months, exceeding the World Health Organization’s recommended timeframe of initiating ANC within the first 12 weeks of pregnancy. Key sociodemographic and behavioral factors significantly influenced the timing of ANC initiation, including age, education level, media exposure, parity, wealth status, desire for more children, place of residence, and religion. These findings underscore the critical need to address inequities in ANC access and utilization, particularly among disadvantaged groups such as women with lower income, education, or are not exposed to media.

The study highlights the need for targeted interventions to promote early antenatal care ANC visits in Kenya, addressing key predictors such as education, media exposure, and wealth disparities. Efforts should focus on empowering women with no education, increasing access to health information through media, and reducing financial barriers for low-income families. Special attention is needed for older women, those with high parity, and women with unintended pregnancies, as they are less likely to seek timely ANC. Additionally, culturally tailored strategies should address religious influences to improve ANC timing and overall maternal health outcomes.

## Electronic supplementary material

Below is the link to the electronic supplementary material.

Supplementary Material 1

## Data Availability

Information is available online and can be found at www.measuredhs.com.

## Abbreviations

AIC: Akaike Information Criteria
AHR: Adjusted Hazard Ratio
ANC: Antenatal Care
BIC: Bayesian Information Criteria
CI: Confidence Interval
EAs: Enumeration areas
KDHS: Kenyan Demographic health survey
PH: Proportional Hazard
WHO: World Health Organizations
