# Supplementary material for: Time to first antenatal care visit and its predictors among women in Kenya: Weibull gamma shared frailty model (based on the recent 2022 KDHS data)
Source: BMC Pregnancy Childbirth. 2025 Jan 22;25:50. doi: 10.1186/s12884-025-07178-y (PMC11752801; doi:10.1186/s12884-025-07178-y)
Supplement: Supplementary file 1 — Supplementary Material 1 [file 12884_2025_7178_MOESM1_ESM.docx]

Supplemental file 1. Akaike Information Criterion (AIC) and Bayesian Information Criterion (BIC) for comparing the best-fitting shared frailty model in the analysis of predictors of time to the first ANC visit among women in Kenya (Number of observations = 10,027)

| **Distribution** | **Frailty** | **ll(null)** | **ll(model)** | **df** | **AIC** | **BIC** |
| --- | --- | --- | --- | --- | --- | --- |
| Gompertz | Gamma | -8144.365 | -7916.893 | 24 | 15881.79 | 16054.9 |
| Exponential | Gamma | -8144.69 | -7917.209 | 23 | 15880.42 | 16046.32 |
| Weibull | Gamma | -8014.912 | -7762.51 | 24 | 15573.02 | 15746.13 |
| Gompertz | Inverse gaussian | -8147.925 | -7917.678 | 24 | 15883.36 | 16056.47 |
| Exponential | Inverse gaussian | -8148.268 | -7917.985 | 23 | 15881.97 | 16047.87 |
| Weibull | Inverse gaussian | -8018.009 | -7762.931 | 24 | 15573.86 | 15746.98 |
